# Supplementary material for: Are carbon emissions trading and green financial instruments synergistic? -Comprehensive quantitative research based on content analysis
Source: PLoS One. 2024 Mar 7;19(3):e0298601. doi: 10.1371/journal.pone.0298601 (PMC10919601; doi:10.1371/journal.pone.0298601)
Supplement: S1 Data — (ZIP) [file pone.0298601.s001.zip › Data/地级市Z_F工作报告.zip!/║╙─╧/╓▄┐┌╩╨/└┤╫╘╣1⁄2╓┌║┼í╛┬φ┐╦╩2╛▌═°í┐_2018.docx]

各位代表：

现在，我代表市人民政府，向大会报告工作，请予审议，并请各位政协委员和其他列席人士提出意见。

一、2017年工作回顾

过去一年，面对跨越发展的新形势新要求，在市委的坚强领导下，全市上下深入学习贯彻党的十九大精神和习近平新时代中国特色社会主义思想，全面落实省委、省政府和市委的各项决策部署，紧盯“三高三优”目标，着力推进“四大攻坚”，奋力实现“四大突破”，经济社会发展稳中向好、稳中求进。全市生产总值2517.03亿元，增长7.9%，高于全省0.1个百分点；一般公共预算收入111.83亿元，增长11.1%，高于全省0.7个百分点；社会消费品零售总额1227.16亿元，增长12.2%，高于全省0.6个百分点；居民人均可支配收入15226元，增长10.2%，高于全省0.8个百分点，实现了产业结构调整、城乡协调发展和生态环境质量更优的预期目标，完成了市四届人大一次会议确定的主要任务。

一年来，广大干部群众思想上同心、目标上同向、行动上同步，一些工作实现了重大突破和进展。

——形成了发展共识。在市委的坚强领导下，相继推出“两违”治理、棚户区改造、临港经济、多式联运等一系列举措，激发了全市上下干事创业的热情，“周口必须加快发展、跨越发展”的共识深入人心，只争朝夕、大干快上的局面正在形成。

——明确了城市定位。按照省委、省政府的部署，基于周口文化厚重、水运发达的优势，通过深入调研、多方论证，确立了以水润城、以绿荫城、以文化城、以业兴城、以港促城，打造“满城文化半城水，内联外通达江海”的中原港城的目标，城市发展有了明确的定位。

——找到了突破路径。周口要弯道超车，实现高质量发展，就必须有一个“牵一发动全身”的产业来带动。我们的优势在水、在港、在运输，建港口、兴物流、聚产业、强服务、优生态、造港城势在必行，打造以“公铁水空”多式联运为基础，内河航运、临港产业、生态城市三位一体融合发展的临港经济新模式应运而生。

——招来了重大项目。新签约亿元以上项目248个，总投资1798.3亿元。港澳创新工业园、万国车世界、太平洋商贸综合体等一批重大项目成功签约，尤其是安钢产能置换项目的落户，即将填补周口在大型重工业项目上的空白。

——破解了“两违”难题。针对“两违”问题突出的现状，重拳出击，短短几个月，全市拆除“两违”建筑300多万平方米，一定程度上消除了长期阻碍城市发展的顽疾。其中，中心城区拆除违法建筑4762处，面积约192.6万平方米；拆除违法用地782处，面积约1772亩。

——加速了棚改进程。实施了棚户区改造，全市征迁3.4万户、967万平方米。特别是中心城区，征迁1.7万户、556万平方米，开工建设安置房2.3万套、面积300.6万平方米。

——决战了脱贫攻坚。3.64万户、14.33万农村贫困人口摆脱了贫困，沈丘有望脱贫摘帽。央视《新闻联播》以《河南特色产业增添脱贫新动能》为题，报道了我市产业扶贫工作的经验。

——壮大了县域经济。鹿邑、项城、沈丘、太康生产总值超250亿元，沈丘、鹿邑、项城、太康、郸城一般公共预算收入超10亿元。食品加工、纺织服装两个千亿级产业集群纳入省打造产业集群计划。太康纺纱规模在全省市县级排名第一，被授予“中国棉纺织名城”称号。项城医药产值突破百亿元大关，跻身全省十大特色产业集群。郸城商务中心区晋级二星级服务业“两区”。

——再创了教育佳绩。高考本科上线人数38732人，上线率56%，稳居全省前列。郸城、扶沟、商水通过国家义务教育基本均衡县评估验收。1234个“全面改薄”项目全面完成。周口科技职业学院通过省级升本验收，我市将再添一所本科院校。

——夺取了粮食丰收。新建高标准粮田91.2万亩，克服阴雨连绵和病虫害等不利因素，实现粮食总产161.7亿斤，为历史第二高产年，继续稳居全省第1位。

一年来，重点做了以下七个方面的工作：

（一）抓临港经济促转型。把临港经济作为转型发展的突破口，引导产业向临港经济集聚、产品向航运市场靠拢。成功举办临港经济发展论坛，研究出台临港经济发展指导意见，规划建设钢铁等五大临港产业园区，全面启动国家级多式联运枢纽试点城市创建工作，产业转型明显加快，三次产业结构持续优化，二三产业占比81.5%，提升1.7个百分点。一是工业转型提速。规模以上工业增加值1048.1亿元，增长8.3%，高于全省0.3个百分点。六大主导产业增长9.8%，拉动工业增长9.3个百分点，对工业的贡献率达112.1%。高成长性制造业增长13.4%，拉动工业增长7.5个百分点，对工业的贡献率达90.7%。高技术产业和高新技术产业分别增长14.3%、15.6%，分别高于工业6个和7.3个百分点。121个重点技术改造项目加快实施。新增院士工作站2家，新建省级重点实验室1家。新认定高新技术企业17家，培育驰名商标3件。二是农业供给提质。土地流转292万亩，建成超千亩的现代农业示范园区22个，培育农业产业化集群40个、市级农业产业化重点龙头企业261家，农民合作社达14922家，家庭农场及种植大户达4600家。规模以上养殖场2336个，畜牧业产值243.7亿元。农产品加工转化率63%。新认证无公害农产品18个、地理标志农产品6个。完成造林9.29万亩。被命名为全国农业综合标准化示范市和“平安农机”示范市。三是服务业水平提升。第三产业增加值893.29亿元，增长10.6%，对经济增长的贡献率44.3%，提升5.4个百分点。第三产业投资741.44亿元，增长10.7%。7家农信社组建农商行。金融机构新增资金投放225.6亿元，是上年的1.6倍。文化旅游业收入115.3亿元。服务业“两区”主营业务收入134.1亿元，增长35.9%。全市电子商务交易额302亿元，增长33.6%；快递业务量完成2795.09万件，增长57.3%。西华、沈丘、淮阳电子商务产业园投入运营，太康、商水被评为国家级电子商务进农村示范县。

（二）抓百城提质上水平。围绕中原港城的发展定位，加快实施百城建设提质工程，城乡面貌持续改善，城镇化率达41.22%，提高1.71个百分点。一是坚持规划引领。完善《周口市城市总体规划（2016～2030）》，编制引黄调蓄工程周边城市设计、沙颍河景观设计等6项城市设计，完成市政基础设施等26项专项规划和高铁片区等5个片区控规。二是加快设施建设。郑合高铁、三洋铁路、周南高速、阳新高速周口段进展顺利，郸沈快速通道全线贯通。周口港物流园区建设步伐加快，沙颍河航道升级改造、涡河航运开发建设有序推进。西华通用机场航展楼建成投用，周口民用支线机场前期选址工作进展顺利。沙颍河城区段综合治理（一期）项目完工。中心城区7条水系治理项目全面开工，八一大道南通商水等一批路网加快推进，汉阳路大闸桥加宽等工程进展顺利。一批公园游园、垃圾中转站、公厕等公共服务设施建成投用。三是推动精细管理。数字化城管平台建成并试运行。出店经营、交通秩序和环境卫生集中整治行动深入开展，城市主次干道机扫率、垃圾无害化处理率分别提高到90%、99.28%。包括10辆双层巴士在内的200辆绿色电动公交车，在中心城区投入运营。中心城区和郸城成功创建省级卫生城市（县城），淮阳荣获国家卫生县城称号。项城、淮阳通过全国文明县城提名城市验收。扶沟、商水、郸城成功创建省级园林县城。

（三）抓精准脱贫补短板。规范贫困户档卡建设，新识别贫困户5947户、15801人。探索出“渔光互补”等扶贫模式，建成市级产业扶贫示范基地110个、标准化“扶贫车间”388个、并网村级光伏电站521个，带动6.69万贫困人口增收脱贫。成立村集体经济合作社3120个，836个有贫困人口的村集体经济收入达5万元以上。建立县乡村三级金融服务平台，完成小额信贷20.16亿元，贫困户获贷率40.45%。推广产业扶贫基地风险保障组合式保险方案，走出具有周口特点的“龙头企业（合作社）+基地+贫困户+金融保险”的路子。整合涉农资金31.54亿元，统筹推进贫困村和非贫困村建设。修建农村公路1421公里，所有贫困村全部完成安全饮水和通动力电工程，实现广播电视户户通。注重把扶贫和扶志、扶技相结合，加大贫困人口转移就业培训，累计解决10.2万贫困劳动力就业问题。累计投入9398.98万元，全面资助17.1万人次建档立卡贫困家庭学生上学，全力阻断贫困代际传递。《人民日报》专题报道了郸城教育扶贫的经验。

（四）抓改革开放激活力。保安公司等市属国有企业公司制改革全面启动，省属企业剥离办社会职能和驻周企业职工家属区“三供一业”分离工作全面完成。“放管服”改革深入推进，全面清理规范行政审批中介服务事项，清理各类证明2004项，取消958项。“三十五证合一”改革持续推进，新登记市场主体68589户，周口豫资投资公司和周口城建投资公司组建运营。全面推行“双随机一公开”机制，实现各级行政执法部门监管全覆盖。医药卫生体制改革持续深化，城市公立医院综合改革全面推开，分级诊疗、家庭医生签约服务试点实施，医联体建设扎实推进。农村土地承包经营权确权登记颁证工作基本完成。不动产统一登记制度规范化建设有序推进。商水入选国家农村能源革命试点县。财税、户籍、供销、国有林场、农垦、盐业、人防和城市执法体制等方面改革进展顺利。在重庆、上海、温州等地举办招商活动，中铝软磁材料、光大垃圾发电等一批项目成功签约。实际利用外资5.36亿美元，到位省外资金486.2亿元。外贸进出口总额61.6亿元，增长19.1%，高于全省8.2个百分点。

（五）抓环境治理优生态。重拳治理大气污染，严格控尘、控煤、控车、控油、控排、控烧，划定区域严格禁燃禁放，“双替代”完成省定任务106%，拆改、关停燃煤锅炉67台，淘汰黄标车6134辆，清理整治“散乱污”企业3188家。PM2.5平均浓度为56微克/立方米，PM10平均浓度为98微克/立方米，分别优于省定目标5微克、3微克，空气优良天数为221天，超过省定目标11天，是全省唯一连续两年全部完成省政府空气质量三项考核指标的省辖市。强化水污染治理，全面落实“河长制”，依法关闭或搬迁禁养区养殖场37个，8个河流目标考核断面达标率93.6%，4个集中式饮用水水源地水质达标率100%。创建省级生态镇5个、省级生态村8个、人居环境达标村2340个。项城被评为河南省美丽乡村建设示范县。

（六）抓社会事业惠民生。重点民生实事和中心城区十件民生实事兑现落实。财政民生支出412.9亿元，占一般公共预算支出的80%。农村劳动力转移就业新增5.9万人，城镇就业新增8万人。项城被确定为全国第三批支持农民工返乡创业试点市，淮阳、商水被评为河南省返乡创业示范县。完成中心城区“八校两园”建设项目。15类国家基本公共卫生服务项目全面实施。深入实施全民参保登记计划，全面实施困难群众大病补充医疗保险制度。郸城被评为国家级妇幼健康优质服务示范县。全面落实低保、优抚、残疾人“两项补贴”等政策。改造农村危房9000户。建成1438个村级综合性文化服务中心。项城袁寨古民居、商水叶氏庄园、西华女娲城、黄泛区生态旅游区创建为3A级旅游景区。省十三届运动会筹备工作有序推进。成功举办河南省全民龙舟大赛、周口马拉松暨中国慈善马拉松赛。构建立体化社会治安防控体系，严厉打击各类违法犯罪行为，扎实做好信访稳定工作，安全生产和食品药品安全形势平稳，人民群众安全感和满意度不断提升。

（七）抓作风转变提效能。坚持把党的领导贯穿政府工作始终，增强“四个意识”，严守政治纪律和政治规矩，始终保持正确的政治方向。严格落实中央八项规定精神，切实加强干部作风建设，认真开展干部作风整顿，扎实解决懒政怠政、政令不通、推诿扯皮、效率低下等问题。各级各单位围绕中心、站位全局，攻坚克难、主动作为，形成了争先恐后、力争上游的良好局面。市发展改革委积极开展对接工作，争取项目259个、资金25.5亿元；市投资集团多次赴北京、上郑州，短短几个月内融资近200亿元，确保了重点项目的有序推进。加强行政监察和审计监督，政府系统廉政建设深入推进，政府的治理能力和水平不断提升。

一年来，我们全面执行市人大及其常委会的决议和决定，自觉接受人大工作监督、法律监督和政协民主监督，认真办理人大代表议案、建议和政协提案，支持民主党派、工商联、无党派人士参政议政。加强国防教育，坚持军民融合发展，沈丘成功创建全国双拥模范城，周口和西华、扶沟、郸城、淮阳成功创建省级双拥模范城（县）。妇女儿童、残疾人、红十字、慈善等事业健康发展。统计、海关、公积金、史志、档案、气象、地震、民族宗教、外事侨务、人民防空、社科研究、应急救援等各项工作均取得新的成绩。

各位代表！

过去的一年，既是全市上下务实重干、敢于担当、加压奋进的一年，也是县域经济大发展、城乡面貌大变化、干部作风大转变的一年。这些成效的取得，是习近平新时代中国特色社会主义思想科学指引的结果，是省委、省政府和市委正确领导的结果，是人大工作监督、法律监督和政协民主监督的结果，是全市广大干部群众苦干实干、奋力拼搏的结果。在此，我代表市人民政府，向奋战在全市各行各业的广大劳动者，向各级人大代表、政协委员，向各民主党派、工商联和无党派人士、各人民团体和离退休老同志，向中央、省驻周单位的同志们以及驻周部队指战员、武警官兵、政法干警，向所有关心、支持、参与周口建设和发展的朋友们，表示衷心的感谢和崇高的敬意！

各位代表！

回顾过去的一年，我们也清醒地认识到，经济社会发展中还面临不少困难和问题，特别是面对高质量发展的要求，我们发展中不平衡、不充分的问题还较为突出。一是发展基础不牢固。我们还有24.37万贫困人口，人均GDP只有28630元，仅相当于全国的1/2、全省的2/3。城镇化率低于全国17.3个百分点、全省8.94个百分点，城乡居民人均收入差距达2.39倍，城乡之间发展不平衡。二是产业结构不优化。三次产业比重为18.5∶46.0∶35.5，农业占比偏高，传统种养业比重大；传统工业占比高，高新技术产业比重小；服务业层次低，拉动作用不强。三是增长动能不充足。固定资产投资增速低于全省0.5个百分点，经济带动能力不足；消费结构不优，经济支撑力较弱；出口多以低端代工产品为主，经济贡献率偏低。科技创新能力不强，新产业新模式新业态培育不够，难以形成较为强大的增长动力。四是民生保障不到位。脱贫攻坚任务艰巨，环境治理压力较大，就业、教育、卫生、住房等公共服务水平有待提升。中心城区民生实事的个别项目没有按时间节点推进。同时，个别政府工作人员能力、素质和作风还不适应新形势、新任务、新要求，在不同程度上还存在着不负责任、不愿担当、效率不高、落实不够等问题。对此，我们将直面正视、高度关注、采取措施、切实解决，决不辜负人民的期望和重托！

二、2018年工作安排

2018年是贯彻党的十九大精神的开局之年，是改革开放40周年，也是决胜全面建成小康社会、持续实施“十三五”规划和全面开启建设中原港城新征程的关键一年，做好今年的工作意义重大。

今年政府工作的总体要求是：以习近平新时代中国特色社会主义思想为指导，认真贯彻落实党的十九大和中央、省委、市委经济工作会议精神，坚持稳中求进工作总基调，坚持新发展理念，紧扣社会主要矛盾变化，按照高质量发展的要求，统筹推进“五位一体”总体布局和协调推进“四个全面”战略布局，围绕“三高三优”和打造“满城文化半城水，内联外通达江海”中原港城的目标，坚持以供给侧结构性改革为主线，以临港经济为抓手，统筹推进稳增长、促改革、调结构、惠民生、防风险各项工作，持续推进“四大攻坚”，奋力实现“四大突破”，促进经济社会持续健康发展。

综合考虑发展趋势和工作任务，建议2018年全市经济社会发展的主要预期目标为：生产总值增长8%左右，固定资产投资增长10%左右，社会消费品零售总额增长12%左右，一般公共预算收入增长8.5%，税收占一般公共预算收入比重65%以上，进出口保持平稳增长，居民收入与经济增长同步，城镇新增就业6万人以上，城镇化率提高1.5个百分点以上，居民消费价格涨幅3%左右，节能减排、环境保护等约束性指标完成省定目标。

做好今年工作，完成上述目标任务，需要我们坚定信心、发挥优势，抓住机遇、应对挑战。从全球视野来审视，建设开放型经济已成为大趋势，大合作、大联动的增长模式逐步成为全球经济治理的共识。世界经济呈现回暖向好态势，美国、欧盟、日本等主要经济体有望继续复苏，国际权威分析机构相继调高今年经济增速预期。从国家层面来把握，中央紧紧抓住全球经济联动大势，提出高质量发展的要求，在开放格局重构、区域布局调整、空间结构优化、动能积蓄发力等方面进行了战略部署。通过“一带一路”、中原城市群、乡村振兴等一系列战略，使东部产业转移加速、中西部崛起加速、新型城镇化加速，给我们带来了更多更大的发展机遇。从全省角度来分析，省委、省政府紧扣中央的战略部署，在积极打造中原城市群和河南自贸区的同时，提出“多极支撑、多点带动”战略，将周口纳入中原城市群核心发展区，列入全省首批百城建设提质工程试点城市，列入创建国家级多式联运枢纽试点城市。这些不仅为我们加快城市建设和临港经济发展提供了支持，更从战略上，让周口从腹地变前沿，成为河南具有区位优势、资源优势、交通优势的经济发展新高地、新引擎、新的增长极。从我市情况来衡量，在全国全省的经济发展布局下，作为郑合高铁经济轴带的重要节点城市、对接长三角的桥头堡和豫货出海口，周口战略地位更加凸显，发展临港经济正当其时。尤其是国家级多式联运枢纽试点城市的创建、安钢产能置换项目的落户，必将加快推动周口迈入工业化、城镇化的新时代。综合判断，我们正处在大有可为的历史机遇期，只要坚定决心、提振信心，务实重干、苦干实干，就一定能完成预期目标，再创新的辉煌！

新时代新任务，新目标新征程。按照市四届人大一次会议和市委四届六次全会要求，为保持工作的连续性，今年政府工作的重点总体上仍然是，持续推进转型发展、精准脱贫、环境治理、深化改革“四大攻坚”，奋力实现城乡规划与建设管理、开放招商与项目建设、棚户区改造与“两违”治理、社会事业与民生改善“四大突破”。同时，根据现阶段的工作重点，将着力做好以下八个方面的工作：

（一）着力发展临港经济。把临港经济作为跨越发展的新引擎，全面发动、加快建设，完善设施、推进项目，吸引更多要素集聚，带动更多产业转型，为发展提供更强动力，为增长蓄积更大动能。

进一步完善发展规划。紧扣“一带一路”和“三区一群”，编制好临港经济发展规划。以周口港为中心，以商水、项城、沈丘、淮阳、西华等港口为支撑，科学规划港口功能。统筹考虑码头、园区、物流、文化等要素，认真编制港口岸线总体规划等各类专项规划，扎实做好“钢铁园区、口岸功能区、装备制造园区、保税园区、物流园区”五大临港产业园区的规划设计。对沙颍河产业进行布局，形成产业隆起带，打造周口特色发展黄金轴线。

进一步建设物流设施。以创建国家级多式联运枢纽试点城市为抓手，科学布局和建设货运枢纽，加快公路、铁路、水路、航空的无缝衔接，着力构建“公铁水空”四位一体的多式联运网络。加快推进沙颍河周口至省界航道升级改造，建成中心港中部作业区8个挖入式港池、47个千吨级泊位，尽快建设集装箱、钢材、粮食等专用码头以及公共泊位、锚地、堆场、岸电等设施，提高配套设施的现代化水平，打造绿色港口、智慧港口。加快推进大广高速港区互通立交、进港铁路专用线、港区公路建设。深化与郑州航空港及沿线港口的合作，建立“空水”联运平台，推进集装箱运输、冷链物流、跨境电商等产业发展。依托现有的港口和18家航运企业，组建周口港航集团。

进一步加快项目组装。加快安钢产能置换项目落地，争取今年上半年一期开工。开工建设粮食产业园，加快葛洲坝兴业再生资源、蓝城特色小镇等项目建设，推进食品加工、装备制造等产业升级。深化与河南物资集团合作，规划建设周口国际港、跨境电商产业园、云数据产业园等项目。

进一步强化要素保障。发挥市投资集团平台融资作用，创新融资模式。加强与豫资公司、省农开公司、省土投公司以及国开行、农发行等金融机构合作，拓宽融资渠道。出台优惠政策，对符合临港经济发展规划的项目，在资金、土地等方面给予倾斜。加大临港产业专业人才的引进和培养力度。持续办好中国临港经济论坛，加强同上海港、河海联运港际合作联盟等机构的协作，不断提升周口内河航运的知名度。

（二）着力推进转型升级。紧盯高质量发展的要求，全面提升传统产业，加快培育新兴业态，打造更多经济增长点。

提升传统产业。认真落实重点产业转型发展“1+7”专项方案，促进主导产业做强做大。抓集群。持续实施“百千亿级产业集群培育”行动计划，积极打造3个千亿级和3个500亿级集群，做好“补链、延链、强链”。实施“一县一主业”集群发展计划，延伸产业链条，推动错位发展。提升产业集聚区建设水平，推动晋星升级。支持市经济开发区争创国家级开发区。提速服务业“两区”发展，积极培育专业服务、特色消费、文化体验等新业态。抓创新。加大科技创新力度，以高端化、智能化、绿色化、融合化为方向，持续实施“三大改造”，推进工业企业提质增效。整合现有创新平台资源，在重点企业、产业基地、产业园区加快组建一批实验室和研发中心，力争再认定一批省级研发平台。建立政府引导基金和大学生创业种子基金。引进一批科技创新领军人才和团队，完善落实资金、住房等方面的优惠政策。抓培育。加快企业战略重组，以30户重点企业、30户高成长性企业为载体，通过兼并重组等方式，推动企业做强做大。利用产业集群优势，加快培育医药产业集团、食品产业集团、纺织产业集团。加强培训，分类指导，重点培育，积极助推企业上市。抓质量。持续实施增品种、提品质、创品牌专项行动，组织开展质量奖评选活动和对标达标专项行动，加快推进精品制造、品牌制造。大力弘扬工匠精神，引导和支持企业导入先进管理模式，打造“百年老店”。抓服务。持续抓好产销、银企、用工、产学研“四项对接”。建立重点企业、重点项目融资信息对接清单，定期组织银企对接活动。持续开展“千名干部帮千企”活动，派驻首席服务员，着力解决企业发展中的困难和问题。同时，加快构建“亲”“清”政商关系，加强对民营企业的产权保护，全面弘扬企业家精神，努力打造一支现代化企业家队伍。

培育新兴业态。壮大“金融+”。积极引进银行、保险、证券等机构落户，做好郑州银行、浦发银行入驻周口的对接工作，争取年内开业。全面完成农信社改制组建农商行工作，力争年内村镇银行实现县域全覆盖。发展多层次资本市场，设立产业投资基金、创业投资基金等融资平台，助力企业发展。提升“文化+”。深度挖掘伏羲、老子、姓氏、农耕、女娲、渡口、漕运、民俗等文化资源，整合吃住行游购娱等要素，高标准编制全域旅游发展规划。在进一步打造好太昊陵、关帝庙、老子故里等旅游景点的基础上，充分对老工厂、老农场、老干校、老铁路等资源进行改造提升，打造新业态，留下历史印记和符号。进一步挖掘传统文化资源优势，积极打造特色小镇、观光农业、乡村民宿等新模式。加强对非物质文化遗产、文化名镇名村、老街区等保护传承和开发利用。规划建设桥梁、内河航运、支前等博物馆，着力打造博物馆文化。支持对民间博物馆、收藏馆、艺术馆、非遗传承所等机构的培育建设。充分挖掘杂技、书画等资源，大力发展休闲娱乐、工艺美术等创意产业，加快培育创意园区、企业和品牌。加快推进临港产业游、水上巴士等新业态发展，谋划建设临港文化旅游小镇。培育“网络+”。加快互联网在制造业、服务业等领域应用，推动互联网医疗、互联网教育、互联网体育等新业态发展。大力发展电子商务，积极引进和培育龙头电商，全面推进电子商务进农村、进社区、进企业。深化云计算、大数据、物联网的应用，积极融入国家（郑州）大数据中心试验区，加快智慧城市建设。

（三）着力提升城市品质。紧抓百城建设提质机遇，坚持以水润城、以绿荫城、以文化城、以业兴城、以港促城，致力于“大干大建出形象、上档升级提品质”。

规划先行重统筹。突出规划先导地位，做到先规划后建设、不规划不建设。统筹好控规、专项规划、城市设计同城市总规之间的协调，综合考虑产业转型、文化旅游、基础设施等因素，实现一体化均衡协调发展。完成城市总规报批和各专项规划的编制工作，实现城市控规全覆盖。加快推进中心城区和淮阳、商水、项城、西华等组团城市规划对接。

加快建设出形象。一手抓老城区改造，推进城市“双修”，方便群众生产生活；一手抓新城区建设，加快项目推进，形成“塔吊林立”的大开发、大建设局面。水系打造上，加快推进中心城区7条水系治理和3600亩的引黄调蓄水面建设，提升改造600亩文昌生态湖，开工建设沙颍河城区段治理（二期）工程，启动中心城区与组团城市水系连通工程，配套新建提升一批桥梁。园林绿化上，实施“一带四林百园”工程，沿环城高速两侧建成300米的生态林带，加快推进5000亩森林公园、6000亩建业绿色基地建设，改造提升2000亩文昌生态园为植物园，启动铁路主题公园建设，新改建100个街头游园。片区开发上，以引黄调蓄周边和高铁片区为重点，加快公共基础设施建设，加强宣传推介，吸引国内外知名金融、地产和文化休闲品牌入驻，打造新区的靓丽名片。启动城市规划馆、文化综合馆等项目建设。基础设施上，加快推进周口东、周口北2个高速出入市口改造升级；完成文昌大道、迎宾大道、周口大道等精品道路的改造提升；实施中原路打通，中州大道、八一大道、大庆路北延，七一路东延，富民路南通商水，开元大道东延港区、西延富民路，武盛大道南延至宁洛高速等工程。按照城市道路标准，改造提升周项、周淮道路，加快文昌大道东延至郸城和中州大道北延至西华高铁站工程，启动周口至太康快速通道前期工作。在中心城区车流人流较大区域和路段，建设城市立交桥和人行过街天桥。加快推进东区污水处理厂、沙南污水处理厂扩建工程和中心城区集中供热工程。新改建100座公厕。着力抓好老旧小区和背街小巷改造，建设一批农贸市场、停车场等公共服务设施。棚户区改造上，持续实施棚户区改造三年计划，中心城区年内完成4.3万户、898万平方米征迁任务，确保2020年完成7万户20万人、183个社区（城中村）、近1800万平方米的棚改任务。启动实施26个安置区、9.2万套安置房建设任务。中心城区加快推进贾鲁嘉园、建设嘉园等安置区建设，确保24个月完成18个安置区、6.98万套安置房建设任务，让搬迁群众住上房、住好房。同时，探索“资源资产化、资产资本化、资本市场化”的路子，拓宽融资渠道，借资发展。节约集约用地，清理闲置土地，做好土地储备，搞好土地开发，实现土地收益最大化。

精细管理提品位。贯彻实施《周口市城市市容和环境卫生管理条例》，深入推进“五城联创”，开展综合执法，持续开展对出店经营、环境卫生、交通秩序的整治。继续保持对“两违”的高压态势，加大巡查排查力度，对存量“两违”分期分批拆除，做到标准不降、力度不减；对新增“两违”零容忍，做到发现一起、拆除一起，坚决维护规划的权威性。强力推进中心城区亮化美化工程，进一步扮靓城市。落实“路长制”，实施物业管理条例，逐街道、逐小区解决群众反映强烈的问题，提升城市形象。

同时，引导、支持各县市加快城市基础设施建设，提升城市管理水平，不断完善产业功能、服务功能和居住功能；加强规划指导和政策资金倾斜，着力打造一批工业强镇、商贸重镇、旅游名镇和特色小镇。加快区划调整步伐，认真落实户籍改革和居住证制度改革政策，推动农业转移人口市民化，稳步提升新型城镇化水平。

（四）着力加快项目建设。把项目建设作为经济发展的主战场，充分发挥项目支撑作用和开放带动作用，积蓄发展后劲。

狠抓招商引资。强化抓招商就是抓发展的理念，坚持大招商、招大商。丰富招商活动。积极参加国家、省举办的重大招商活动，在珠三角、长三角、京津冀等重点地区开展专题招商，举办“知名周商周口行”活动，承接一批项目。突出招商重点。制定临港经济专项招商方案，力争万邦物流、远大装备制造等项目在产业园区落地。充分发挥产业图谱作用，瞄准世界和国内500强企业、中央企业、省管企业和关联度高的企业，精准对接项目。创新招商模式。推进集群式招商，放大信息、技术、资金、政策和资源的集聚效应。深化与中央企业、省管企业合作，推行股权招商、并购招商。发挥工商联、商会和企业主体作用，开展以商招商。支持各类产业园区联合行业协会，开展整体招商。利用好投融资平台，发挥政策性银行作用，探索建立产业引导资金和科技孵化创投基金，撬动更多社会资本支持周口发展。务求招商实效。坚持旬通报、月排名、季督查、年总评，进一步完善招商推进和激励机制，不断推动招商工作取得实效。

狠抓项目建设。紧紧扭住项目建设不放松，着力抓开工、抓投产、抓进度、抓形象，努力形成储备项目源源不断、开工项目铺天盖地、在建项目热火朝天、竣工项目接二连三的生动局面。统筹谋划重大项目。围绕临港经济、百城提质等，谋划一批全局性、引领性的重大项目。加快推进周口民用支线机场项目前期审批、选址工作。积极谋划洛周商城际铁路、濮潢铁路等项目。积极争取项目资金。认真研究国家宏观调控和省里的产业政策、投资政策，加大对中央预算内投资项目、中央专项建设基金项目和省专项资金项目的争取力度。滚动实施“5818”扩大有效投资行动，规范有序推进PPP项目。强力推进重点项目。突出抓好58个省重点项目和313个市重点项目，尤其是100个中心城区“四个一批”项目。加快推进乐普药业固体制剂改扩建、金丹聚乳酸、合益纺织、闽商食品产业园、金华夏装配式建筑等项目建设。加快推进西华通用机场、鹿邑通用机场、郑合高铁、三洋铁路、周南高速周口段建设，开工建设阳新高速周口段，改造升级一批干线公路，加快建设宁洛高速开发区互通立交。加快推进华耀城、昌建、天明城等城市综合体建设，启动万达广场以及恒大、建业城市综合体建设。周口燃气热电项目、燃煤发电项目实现双机组投产运行。开工建设周口至驻马店东500千伏线路工程，解决周口电网电源来源方向单一问题。

狠抓服务保障。强化项目责任制，坚持一个项目、一名领导、一班人马、一套机制、一抓到底“五个一”制度。坚持重点项目协调推进周例会机制，切实解决项目推进中存在的土地、资金等方面的具体困难和问题。继续开展观摩评比、集中开工及“百日会战”等活动，建立定期督查机制。推行“一站式”服务，规范行政行为，提高办事效率，为企业松绑减负。

（五）着力实施乡村振兴。按照产业兴旺、生态宜居、乡风文明、治理有效、生活富裕的总要求，科学编制实施乡村振兴战略规划，探索出一条有周口特色的乡村振兴之路，推动实现农业强、农村美、农民富。

深入推进精准脱贫。当前，脱贫攻坚已进入系统发力、集中攻坚的关键阶段，我们将始终坚持精准扶贫、精准脱贫，真正把扶持对象、项目安排、资金使用、措施到户、因村派人、脱贫成效“六个精准”落到实处，确保如期完成目标任务。重产业。加大产业扶贫示范基地建设，再打造市级产业扶贫示范基地100个以上，实现所有贫困户产业扶贫全覆盖。抓好标准化“扶贫车间”建设，带动更多贫困户就近就业增收。推广“渔光互补”和“爱心超市”等产业扶贫项目，拓宽增收渠道。加快发展村级集体产业，确保贫困村有稳定的经济收入。强化金融扶贫，采取有力措施，加快投放进度，确保完成年度任务。重培训。在扶贫中突出扶智、扶技，利用雨露计划、阳光工程等政策措施，加强对贫困人口的职业技能培训，提高其创业就业能力和脱贫致富本领。把扶贫与扶志相结合，努力变“外部输血”为“自身造血”，激发贫困人口的内生动力。重设施。统筹整合涉农资金，持续加大投入，统筹推进贫困村和非贫困村的基础设施建设，实现通村道路、标准化卫生室、综合性文化服务中心等全覆盖。重责任。认真落实转移就业、社会保障、特殊救助、医疗卫生、教育培训等方面扶贫政策，夯实行业帮扶责任。强化市县主体责任、部门协同责任、乡村包干责任、驻村帮扶责任，建立层层压实的责任体系。完善督查巡查方式，严禁弄虚作假、搞数字脱贫。

大力发展现代农业。树立大食物观。坚持藏粮于地、藏粮于技，在坚持粮食安全责任制、稳定粮食产量的基础上，进一步优化产能。以市场需求为导向，按照“四优四化”要求，持续调优种植结构，优质强筋小麦、优质花生、优质大豆、优质芝麻分别达到120、160、100、60万亩。进一步扩大生猪、肉鸡、牛羊及奶业等优质畜产品生产基地规模，积极创建省级畜产品质量安全示范市。支持扶沟依托蔬菜产业优势，积极创建国家级现代农业产业园。树立大农业观。探索发展农业生产性服务业，通过土地托管、土地流转、入股分红等形式，发挥产业链服务作用，充分释放农业生产的新动能。实施农产品加工业提升行动，依托益海、鲁花、邦杰等龙头企业，大力培育农业产业化集群。

积极打造三产融合示范基地，创建国家农村产业融合发展示范园。加强农业科技投入，强化农业技术推广和成果转化。树立大市场观。实施农产品品牌战略和食品安全战略，创建特色农产品优势区，既让农产品种得好卖得好，又让农民增产增收。树立大生态观。科学施用化肥、农药，以现代农业园区为平台，逐步建立绿色低碳循环的农业产业体系，探索现代农业发展新模式。

加快改革释放活力。深入推进农村土地制度改革，巩固深化农村土地承包经营权确权登记颁证成果，完善承包地“三权”分置制度。深化农村集体产权制度改革，探索盘活闲置农房和宅基地的办法，推动资源变资产、资产变股权、资金变股金、农民变股东。推进农村信用服务体系建设，激活农村产权价值，激发农村金融活力。

注重培育职业农民。大力培育新型农业经营主体，发展多种形式适度规模经营，推动小农户、大生产和大市场的有机衔接。充分发挥我们在外务工人员、创业成功人士多的优势，鼓励支持他们返乡创业，激励各类人才到农村广阔天地大显身手，让农民这一职业成为体面的职业。

全面开展乡村治理。落实好农村人居环境整治三年行动方案，深入开展“百日行动”“六村共建”，加快推进“厕所革命”，重点抓好农村垃圾清理、文明乡风提升、农村绿化美化三项工作。每村配备专职保洁人员，探索建立农村卫生保洁的长效机制，努力实现环境美、田园美、村庄美、庭院美。畅通零售、电商渠道，改善农村消费环境。加强农村精神文明建设，创建文明村镇、文明家庭。持续推进农村基层组织建设，强化“四议两公开”决策机制，健全自治、法治、德治相结合的乡村治理体系。

（六）着力深化改革攻坚。改革是激发动能的重要举措，也是推动高质量发展的关键，我们将紧盯重点领域，把各项改革部署落实到位。

全面推进国企改革。按照《周口市深化国有企业改革总体方案》，针对市属国有企业改革不彻底、不到位的现状，坚持分类指导，一企一策，加快推进。抓住“僵尸企业”处置这个“牛鼻子”，着眼“五个全面”既定目标，突出企业产权、组织、治理“三大结构”改革，积极发展混合所有制经济，加快建立现代企业制度。加强国有资产监管，防止国有资产流失，实现国有资产保值增值。

全面深化“放管服”改革。在“放”的方面，再精简一批审批事项、审批中介服务事项，加快推进“三十五证合一”改革，实行市场准入负面清单制度。在“管”的方面，完善事中事后监管制度，全面实施“双随机一公开”监管方式。在“服”的方面，深入实施模拟审批，加强“互联网+政务服务”建设，全面推行行政权力事项在线办理，实现“平台之外无审批”。积极实施企业登记全程电子化。健全预算公开制度，继续落实“营改增”政策。持续推进涉企资金基金化改革，重点抓好产业集聚区发展投资基金和现代农业发展基金的参股设立和运营，为产业发展提供资金支持。

全面防控风险隐患。突出“降杠杆、治乱象、抓规范、强监管”四个重点，防范化解不良资产、政府债务、影子银行、非法集资等风险隐患，确保不发生区域性、系统性风险。着力抓好社会信用体系建设，落实行业监管部门主体责任，有效化解不良资产。严控政府债务规模，全面开展政府债务审计，健全融资机制，严禁突破省定限额举借新债。强化企业风险意识，引导企业加强财务管控，优化债务结构，降低财务杠杆。大力发展普惠金融，降低企业融资成本。严厉打击非法集资，切实防范社会领域风险。

同时，进一步深化农垦、供销、国有林场、文化教育、医疗卫生、城管、环保、盐业等方面改革。

（七）着力做好环境保护。紧盯省定目标，坚持全民共治、源头防治、科学防治、标本兼治，深入推进大气、水、土壤污染防治，着力打造天蓝、水净、地绿的美丽周口。

加快推进蓝天工程。坚持长短结合、铁腕治污，突出重点区域、重点企业、重点时段管控，强化“六控”措施落实。加快推进35蒸吨以下燃煤锅炉拆除改造，中心城区安装12套汽车尾气遥感监测设备。加大散煤治理力度，城区全面禁止燃烧散煤，农村逐步推广使用清洁型能源。实施小散乱污动态清零等专项行动，强化对扬尘、工业、秸秆焚烧等污染源的综合防控，积极做好重污染天气应对工作，努力让蓝天白云常在。

加快推进碧水工程。全面落实“河长制”，坚持全域治理、系统治理、源头治理，强化企业排放监管。加强饮用水水源地和重点流域水质保护，全面开展洺河、涡河等河流整治，确保八个地表水考核断面达到省定目标值，四个集中式饮用水源地水质达标率100%。开展黑臭水体全面清除专项行动，年内中心城区建成区消除黑臭水体，县城建成区完成截污纳管工作，努力让涓涓清水常流。

加快推进土壤污染防治工程。坚持最严格的耕地保护制度，加强农业面源污染防治，严格危险废物环境监管，开展土壤污染治理和修复。综合治理畜禽粪便，综合利用秸秆资源，控源治理过量使用化肥、农药，促进土壤资源永续利用，努力让万顷良田常肥。

加快推进林业生态建设工程。着力抓好森林城市、森林廊道、森林田园、森林社区、森林乡村等工程，加快实施国家储备林项目，新增造林绿化面积11.2万亩，创建一批省级森林城市、绿化模范乡镇，积极创建全国绿化模范城市，努力让周口大地常绿。

（八）着力改善社会民生。坚持以人民为中心的发展思想，压缩一般性开支，挤出更多财力用于民生改善，办好省、市民生实事，让人民群众有更多获得感、幸福感。

大力促进就业创业。落实就业创业扶持政策，推动大众创业、万众创新。突出做好高校毕业生、农村转移劳动力、城镇困难人员、退役军人等就业工作，妥善解决下岗分流职工再就业问题。城镇登记失业率控制在4%以内。

完善社会保障体系。全面实施全民参保计划。推动城乡居民基本医保与大病保险制度有效衔接，全面落实困难群众大病补充医疗保险制度。进一步完善城乡居民基本养老保险制度，完成机关事业单位养老保险制度改革。继续提高城乡低保、基础养老金、特困人员生活费标准，落实好困难残疾人生活补贴和重度残疾人护理补贴制度。加强农村低保与扶贫政策的有效衔接，提高低保精准施保水平。

全面发展社会事业。实施第三期学前教育三年行动计划，新改建一批幼儿园，逐步缓解“入园难”问题。持续实施农村“全面改薄”、城镇学校扩容提质和教育信息化三大工程，推动义务教育城乡一体化均衡发展，力争沈丘、项城、鹿邑通过国家义务教育均衡发展达标验收。持续打造高中教育品牌。继续实施中心城区教育振兴工程。推进职业学校提级提质发展，支持周口科技职业学院升本后加快发展，支持联营职业学校晋升大专，支持周口高级技工学校晋升技师学院。实施“校长工程”，全面加强教师队伍建设。广泛开展全民健身活动，助力健康周口建设。加快医疗联合体建设和医养结合工作，继续实施好分级诊疗、家庭医生签约服务和卫生计生服务体系建设。加快推进市中心医院新区医院、市中医院东区分院等重点项目建设。持续实施文化惠民工程，开工建设周口大剧院、烈士陵园和24小时智慧图书馆。加快公交枢纽场站和充电场站建设。

精心筹办省运会。省十三届运动会是对周口的一次大考，我们将集全市之智、举全市之力，强化部门联动，强化协作配合，强化责任落实。在场馆建设上，提效率、抢进度、赶工期，确保3月底前全部完工；在开闭幕式、接待服务、安全保卫上，查漏补缺、完善提升。采取“政府引导、市场运作、社会赞助”的办法，做好文化交流、经贸洽谈等活动，努力把省十三届运动会办成一届富有特色、精彩纷呈的盛会。

推进社会治理创新。持续开展示范性社区建设。加强法治宣传教育和法律服务，落实信访维稳工作责任制。严格落实安全生产责任制，推进安全发展示范城市创建，坚决遏制重特大安全事故发生。强化食品药品安全治理，加快推进国家食品安全城市创建。持续深化平安周口创建，加强社会治安防控体系建设，深入开展扫黑除恶专项斗争，不断增强群众安全感、提升群众满意度。

加强国防教育、国防动员，支持驻周解放军、武警部队、预备役部队、民兵和人民防空建设，推进军民融合发展，深入开展双拥共建，积极争创全国双拥模范城。全面贯彻党的民族政策和宗教工作基本方针，规范宗教行为，促进民族团结、宗教和睦。支持工会、共青团、妇联等人民团体更好发挥桥梁纽带作用。加强统计、海关、公积金、史志、档案、气象、地震、外事侨务、社科研究、应急救援等工作。

三、全面加强政府自身建设

人民满意是我们的最高追求。2018年，面对千万人民的期盼和艰巨繁重的任务，我们必须切实加强自身建设，全面提高施政能力和服务水平，努力建设人民满意政府。

旗帜鲜明讲政治。坚持党的集中统一领导，把政治建设放在首位，以习近平新时代中国特色社会主义思想为指导，推进各项政府工作。牢固树立“四个意识”，坚定“四个自信”，做到“四个服从”，始终在思想上政治上行动上同以习近平同志为核心的党中央保持高度一致。坚决服从市委的领导，坚决贯彻市委的决策，坚决维护市委的形象。

不忘初心勤为民。我们将不忘初心、牢记使命，始终把人民对美好生活的向往作为奋斗目标，当好千万人民的“长工”。秉承党的宗旨，坚持群众路线，坚决杜绝脱离群众的作风和行为。真正体会群众的求生之艰、求学之难、求医之急、求职之苦，多做惠民之事、多解民生之忧，千方百计让周口人民过上更加美好的生活。

敢于担当尽职责。我们将勇于正视工作中存在的问题，勇于克服工作中遇到的困难，对应该做的事，克服困难也要干；对必须负的责，顶着压力也要担。坚持“实”字当头、“干”字为先，勇于负责、勇于担当，敢想敢干、敢闯敢试，决不瞻前顾后、畏首畏尾，决不回避矛盾、推卸责任，以逢山开路的闯劲、攻城拔寨的拼劲、抓铁有痕的韧劲，推动各项工作落地生根。

务实重干转作风。开弓没有回头箭。周口大建设的序幕已经拉开，社会在关注，群众在期盼。我们将会始终坚持苦干、实干、加油干，做到重大决策一以贯之，决不动摇折腾；重大项目一抓到底，决不半途而废；重大任务一鼓作气，决不无果而终。将以慢不得、坐不住、等不起的紧迫感，雷厉风行，加快节奏提效率，风风火火干工作，夙兴夜寐尽责任，做到立说立行、马上就办，决不等待观望、懒懒散散。持续深化政府系统干部作风大整顿，做到重点工作安排部署到哪里，督查督导工作就跟进到哪里。紧盯不落实的事，倒查不落实的人，该约谈的约谈，该曝光的曝光，该追责的追责，坚决防止表态多调门高、行动少落实差等现象发生，真正让求真务实成为政府工作的主旋律。

依法行政受监督。牢固树立法治理念，增强尊法学法守法用法意识，坚持依宪施政、依法行政，认真做到法定职责必须为，法无授权不可为。坚持科学决策、民主决策、依法决策，落实公众参与、专家论证、风险评估、合法性审查和集体讨论决定等重大决策程序，广泛听取各方面意见包括批评意见。按照“四化三查”要求，严格规范政府行为，打通服务群众“最后一公里”问题。自觉接受人大的法律监督、工作监督和政协的民主监督，认真办理人大代表议案、建议和政协提案。支持民主党派、工商联、无党派人士参政议政。深化政务公开，畅通社会监督渠道，让权力在阳光下运行。

廉洁从政葆本色。严格落实全面从严治党主体责任和“一岗双责”，严格落实中央八项规定实施细则，健全行政权力监督制约机制，强化廉政风险防控。进一步规范招投标市场秩序，提高招投标质量和效果。深入推进“两学一做”学习教育常态化制度化，扎实开展“不忘初心、牢记使命”主题教育，自觉廉洁用权、廉洁修身、廉洁齐家。持之以恒正风肃纪，严肃查处各类腐败，着力营造清朗政风。

各位代表！

新时代赋予新使命，新征程召唤新作为。周口的发展，需要大干；周口的事业，需要苦干；周口的建设，需要实干。2018年注定是周口大干、苦干、实干的一年，让我们大家多当战斗员、少当评论员、不当观察员，全力以赴推动周口加快发展。让我们更加紧密团结在以习近平同志为核心的党中央周围，在省委、省政府和市委的坚强领导下，振奋精神、坚定信心，锐意进取、扎实工作，为决胜全面小康、实现跨越发展、建设更加幸福美丽的新周口而努力奋斗！
